# How Do Plants and Phytohormones Accomplish Heterophylly, Leaf Phenotypic Plasticity, in Response to Environmental Cues

**DOI:** 10.3389/fpls.2017.01717

**Published:** 2017-10-04

**Authors:** Hokuto Nakayama, Neelima R. Sinha, Seisuke Kimura

**Affiliations:** ^1^Department of Plant Biology, University of California, Davis, Davis CA, United States; ^2^Department of Bioresource and Environmental Sciences, Kyoto Sangyo University, Kyoto, Japan; ^3^Center for Ecological Evolutionary Developmental Biology, Kyoto Sangyo University, Kyoto, Japan

**Keywords:** phenotypic plasticity, heterophylly, phytohormones, *Potamogeton nodosus*, *Rorippa aquatica*, *Ludwigia arcuata*

## Abstract

Plant species are known to respond to variations in environmental conditions. Many plant species have the ability to alter their leaf morphology in response to such changes. This phenomenon is termed heterophylly and is widespread among land plants. In some cases, heterophylly is thought to be an adaptive mechanism that allows plants to optimally respond to environmental heterogeneity. Recently, many research studies have investigated the occurrence of heterophylly in a wide variety of plants. Several studies have suggested that heterophylly in plants is regulated by phytohormones. Herein, we reviewed the existing knowledge on the relationship and role of phytohormones, especially abscisic acid, ethylene, gibberellins, and auxins (IAA), in regulating heterophylly and attempted to elucidate the mechanisms that regulate heterophylly.

## Introduction; What is Heterophylly?

**FIGURE 1:**
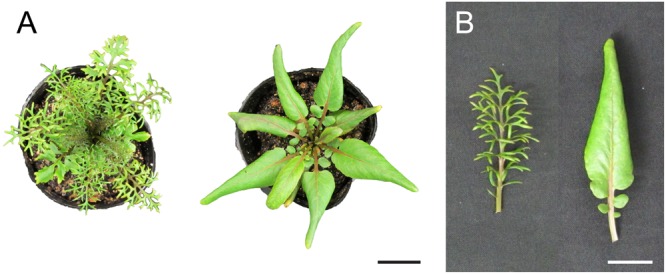
Heterophylly in *Rorippa aquatica*. **(A)** Top view of shoots grown under each condition for a month. Left, at 20°C; right, at 30°C. **(B)** Mature leaf morphology of the seventh leaf. Left, at 20°C; right, at 30°C. Bars = 3 cm in **(A)** and 2 cm in **(B)**.

Plants have the ability to alter their morphology in response to environmental conditions. This phenomenon is known as phenotypic plasticity (Alpert and Simms, 2002; Zotz et al., 2011). Phenotypic plasticity exhibited as leaf form alteration in response to environmental conditions such as light intensity and quality, ambient temperature, and water availability is called heterophylly (**Figures 1A,B**). The original definition of heterophylly was not strictly linked to the environmental control. However, recently, it has been often the case that heterophylly refers to leaf form alteration in response to environmental cues (Anderson, 1978; Goliber and Feldman, 1990; Kuwabara et al., 2003; Wanke, 2011; Sicard et al., 2014). This phenomenon differs from heteroblasty, which refers to conspicuous morphological changes in leaves throughout the lifecycle of plants (Zotz et al., 2011). Additionally, heterophylly differs from anisophylly, which is a special case of dorsiventral shoot symmetry in which leaves inserted on the dorsal and ventral sides of the stem differ in size and shape. Anisophylly is normally coupled with leaf and stem asymmetry and modified phyllotaxis (Dengler, 1999). Therefore, heteroblasty and anisophylly do not include morphological changes induced by environmental stimuli, whereas heterophylly is expressed as the environmentally induced switch between two or more leaf morphologies in the same plant (Zotz et al., 2011).

Heterophylly is exhibited by various land plants including terrestrial and aquatic species (Wanke, 2011; Nakayama et al., 2012). Among angiosperms, heterophylly occurs in diverse taxa. Several studies (Goliber and Feldman, 1990; Kuwabara et al., 2003; Iida et al., 2016) on heterophylly have indicated that this trait has evolved multiple times during plant evolution among various unrelated taxa. In some cases, heterophylly is perceived to be an adaptive mechanism that allows plants to optimally respond to environmental heterogeneity (Palacio-Lopez et al., 2015). Adaptive plasticity hypothesis predicts that plants capable of exhibiting heterophylly in leaf architecture in response to heterogeneous environment are expected to have better fitness compared to other plants. However, there is limited information on the adaptive significance of heterophylly. Moreover, theoretical studies indicate that the acquisition of heterophylly may be constrained by the genetic costs and limits of plasticity (Ernande and Dieckmann, 2004). Hence, it is debatable whether all heterophylly evolved as an adaptive response (Palacio-Lopez et al., 2015).

Heterophylly is the focus of many studies due to its uniqueness (Fassett, 1930). Studies on the molecular mechanisms underlying heterophylly have been published recently (Kuwabara et al., 2003; Nakayama et al., 2014; Sicard et al., 2014). Interestingly, many studies have suggested that various phytohormones are involved in the regulation of heterophylly. Therefore, we considered it worthwhile to review the existing knowledge on the relationships and interactions between heterophylly and phytohormones to gain valuable insight into this phenomenon.

## How Does ABA Regulate Heterophylly?

**FIGURE 2:**
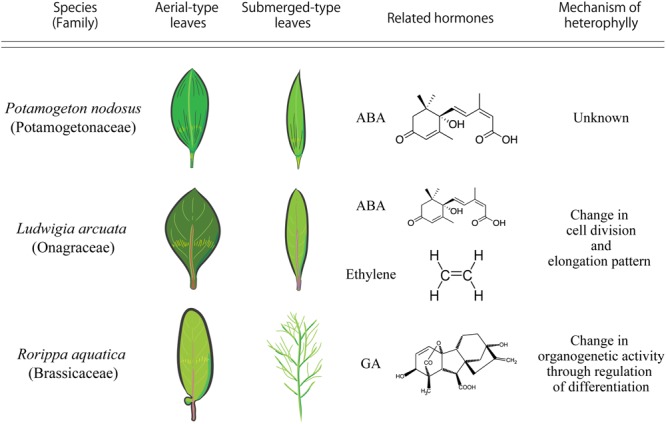
Comparison of heterophylly seen in *Potamogeton nodosus, Ludwigia arcuata*, and *Rorippa aquatica*.

*Potamogeton nodosus* (Potamogetonaceae), an aquatic plant native to Eurasia and North America, exhibits heterophylly in the form of two distinct types of leaves: long narrow submerged leaves and oblong elliptical floating leaves (Anderson, 1978). A report published in 1978 showed that a low concentration of exogenous abscisic acid (ABA) induced floating leaves in *P. nodosus* (**Figure 2**) (Anderson, 1978).

Abscisic acid, a tiny molecule classified as a sesquiterpene, is one of the well-known hormones regulating abiotic stress responses in plants (Vishwakarma et al., 2017). ABA is thought to be synthesized in the vasculature and in the guard cells of the vegetative part of the plant (Boursiac et al., 2013). Interestingly, ABA as a signaling molecule has been reported in a phylogenetically wide range of organisms from cyanobacteria to human (Maršálek et al., 1992; Bruzzone et al., 2007). Some studies have suggested that the ABA pathway is conserved in the green plant lineage (Takezawa et al., 2011). However, little is known about why various kinds of organisms utilize and respond to ABA (Lievens et al., 2017). ABA is involved in controlling growth and development of plants such as leaf abscission, inhibition of fruit ripening, and drought stress response.

*Ludwigia arcuata* (Onagraceae) is one of the well-characterized aquatic plants exhibiting heterophylly. This plant forms narrow leaves under submergence, and round leaves under aerial growth conditions (**Figure 2**) (Kuwabara et al., 2003). Analytical studies of the different developmental stages of *L. arcuata* demonstrated that ABA plays an important role during the change in leaf morphology between submergence and aerial conditions as is also reported in *P. nodosus* (Anderson, 1978). In *L. arcuata*, application of exogenous ABA to submerged shoots resulted in aerial leaf form even under submerged condition (Kuwabara et al., 2003). As described above, a notable feature of ABA synthesis is for drought stress response. Several studies on a variety of plant species have suggested that osmotic stress conditions induce the production of ABA, which acts as a controller in stress response and tolerance of plants (Yamaguchi-Shinozaki and Shinozaki, 2006; Nakashima and Yamaguchi-Shinozaki, 2013) and the accumulated ABA in vegetative tissues induces ABA-responsive gene expression (Goda et al., 2008). These studies substantiate the role of ABA in the regulation of heterophylly, especially in aquatic plants that can sense changes in the surrounding environment, in particular, water level and/or availability, via ABA. Indeed, in addition to functioning as a short-distance signaling molecule, it has been suggested that ABA is a long-distance signaling molecule that is transported from mature leaves to developing leaves to optimize some phenotypes such as stomatal development in response to environmental changes (Chater et al., 2014). Hence, when submerged *L. arcuata* leaves were brought in contact with air, the endogenous levels of ABA increased and this is presumed to initiate and induce heterophylly in *L. arcuata* (Kuwabara et al., 2003). Interestingly, ABA application was sufficient for the formation of the terrestrial leaf form in other heterophyllous aquatic plants also (Kane and Albert, 1987; Goliber and Feldman, 1989; Hsu et al., 2001). Regulation of heterophylly by ABA in many plants is not surprising, since the origin of ABA signaling pathway is thought to be ancient and is conserved in the green plant lineage (Takezawa et al., 2011). Thus, these facts indicate that the ABA signaling pathway can be considered a hotspot in plant evolution to acquire heterophylly, even though this trait is suggested to have evolved multiple times in distant plant species.

## How Does Ethylene Regulate Heterophylly?

Ethylene has a long history as a gaseous phytohormone since its discovery from studies initiated in the late 1800s (Fahnestock, 1858). Subsequently, researchers identified ethylene as the active component of the illuminating gas that affects plant growth and ethylene synthesis by plants was reported in the early 1900s (Neljubow, 1901). Ethylene (C_2_H_4_) regulates many aspects of plant developmental and physiological processes, including seed germination, root initiation, flower and leaf senescence, abscission, fruit ripening, wound response, and defense against diseases (Schaller, 2012). Some studies have shown the relationship between ethylene and heterophylly. In *L. arcuata*, ethylene as well as ABA are known to be the key factors regulating heterophylly; ethylene treatment induced the formation of submerged leaves in this plant (Kuwabara et al., 2003; **Figure 2**). Additionally, endogenous concentration of ethylene was higher in these plants under submergence compared to those under terrestrial conditions (Kuwabara et al., 2003). Developmental and anatomical studies have suggested that the changes in cell division patterns induced by ethylene resulted in leaf form alteration in *L. arcuata* (Kuwabara and Nagata, 2006). Several studies have indicated that ethylene not only regulates cell size, often restricting cell elongation, but also regulates cell division (Iqbal et al., 2017). Ethylene is thought to be synthesized in almost all plant tissues and accumulates in the plant tissues under submergence because solubility of ethylene in water is low (Davis and McKetta, 1960) and it cannot evaporate easily from the submerged plant parts. The increased concentration of ethylene accumulated in the submerged parts of *L. arcuata* is assumed to induce changes in cell elongation and cell division and regulate leaf morphology. Moreover, it is known that ethylene not only acts on ABA metabolism to reduce ABA levels, but also negatively regulates ABA signaling during germination in *Arabidopsis thaliana* (L.) Heynh. (Arabidopsis hereafter) (Gazzarrini and McCourt, 2001). Indeed, ethylene treatment reduced endogenous level of ABA in *L. arcuata* (Kuwabara et al., 2003), suggesting that ethylene regulates heterophylly through suppression of ABA and regulating cell division and elongation. In addition to heterophylly, ethylene is also reported to be involved in submergence responses in deepwater rice (Hattori et al., 2009). The increase in concentration of ethylene in the submerged parts of deepwater rice triggers remarkable elongation of internodes, which have a hollow structure to allow gas exchange with the atmosphere. Moreover, ethylene is known to be involved in development of aerenchyma, which is an intercellular space that acts as a mediator of internal gas exchange and maintains physical strength of tissues (Dengler, 1999). These phenomena are also a type of phenotypic plasticity. These studies indicate that utilization of the ethylene signaling pathway under submergence by plants has evolved multiple times for the regulation of phenotypic plasticity, including heterophylly. Thus, it is likely that the ethylene-related pathway may be a well-used machinery of phenotypic plasticity in aquatic plants, as is the case with ABA.

## How Do Gibberellins Regulate Heterophylly?

Gibberellins (GAs or GA) were first identified in response to the pathogenic fungus *Gibberella fujikuroi*, which causes excessive elongation of the stem in *Oryza sativa* (rice) (Yabuta and Sumiki, 1938). To date, more than 130 GAs have been identified from fungi, bacteria, and plants (Yamaguchi, 2008). GA is indispensable for various kinds of plant processes such as seed germination, stem elongation, expansion of leaf lamina, pollen maturation, and flowering (Sun, 2010). GA is also involved in the regulation of heterophylly. In the North American semi-aquatic plant *Rorippa aquatica*, GA is thought to be a key factor for the regulation of heterophylly (Nakayama et al., 2014); this plant produces deeply dissected leaves under water and simple leaves with smooth margins under terrestrial conditions (**Figure 2**). Leaf complexity of *R. aquatica* is similarly affected by changes in the ambient temperature; deeply dissected leaves develop when plants grow at 20°C, whereas simple leaves with entire margins develop when plants are grown at 25°C (**Figures 1A,B**). A previous study showed that in *R. aquatica*, the expression level of *KNOTTED1 LIKE HOMEOBOX* (*KNOX1*) ortholog changes in response to changes in the ambient temperature. KNOX1 protein is known to regulate GA levels in leaf primordia (Sakamoto et al., 2001). GA concentration in leaf primordia changes in response to the ambient temperature, and exogenous GA application alters the leaf complexity in *R. aquatica*. Similarly, in *Solanum lycopersicum* (tomato), GA promotes differentiation of leaf primordia, and disables transient organogenetic activity in the leaf margins, from which marginal serrations and leaflets arise; thus, GA reduces leaf complexity in *S. lycopersicum* (Yanai et al., 2011). Therefore, heterophylly in *R. aquatica* is thought to be regulated by the alteration of GA concentrations in leaf primordia via the *KNOX1* gene. Developmental studies in *R. aquatica* have indicated that proximal leaflet initiation in leaf primordia is an important factor in determining final leaf form. These studies suggest that the local GA concentration in leaf primordia is important for the regulation of heterophylly in *R. aquatica*. In addition to heterophylly, Arabidopsis mutants, which are insensitive to the GA and defective in its biosynthesis show a delayed appearance of the first adult leaf compared to WT (Chien and Sussex, 1996), suggesting that GA is involved in the heteroblasty. Therefore, GA may be utilized both of heterophylly and heteroblasty. Interestingly, a previous study showed that a single leaf can sense and transmit changes in ambient temperature to newly developed leaves in *R. aquatica* (Nakayama and Kimura, 2015), suggesting that a long distance signal may be generated at a certain developmental stage of leaves. Transmembrane transport of GA in Arabidopsis is reported to be regulated by AtSWEET13, AtSWEET14, and AtNPF2.10/GTR proteins (Kanno et al., 2016). *R. aquatica* belongs to the same family as Arabidopsis and hence the expression pattern and function of many genes are expected to be similar in both the plant species. Similar orthologs may also be responsible for cellular GA uptake in leaf primordia of *R. aquatica*. Although GA can be transported through the phloem (Hoad and Bowen, 1968), the detailed molecular mechanism of the long distance GA transport remains unclear. A better understanding on the long distance GA transport may reveal its role in regulating heterophylly.

## How Do Auxins Regulate Heterophylly?

Auxins play a key role in an extraordinarily wide variety of biological processes in terrestrial plants. For example, auxins are involved in plant growth and development such as abscission, apical dominance, cell division and differentiation, flowering, senescence, and tropic responses (Sauer et al., 2013). Auxin biosynthesis is intricate and multiple pathways have been postulated to explain auxin biosynthesis (Chandler, 2009; Normanly, 2010; Zhao, 2010). Additionally, auxin biosynthetic pathways are differentially regulated in response to environmental stimuli (Tao et al., 2008; Le et al., 2010). In Arabidopsis, auxin is thought to be synthesized throughout the shoot apical meristem (Cheng et al., 2006; Stepanova et al., 2008) and transported with transporter proteins such as PIN1 (Galweiler et al., 1998). Several papers and reviews detail their mechanism of auxin transport (Adamowski and Friml, 2015). The polarization of auxins is indispensable for the initiation of leaf primordia and leaf lamina outgrowth during leaf development (Byrne, 2012; Townsley and Sinha, 2012). First, auxin maxima develop at the tip of the leaf primordia, and are thought to lead to distal growth. The auxin is then transported from the leaf margins and distributed on either side of the midvein; this facilitates leaf lamina outgrowth as a downstream target of the adaxial-abaxial polarity pathway (Scarpella et al., 2010). Additionally, auxins are also involved in vascular patterning in leaves (Scarpella et al., 2010), which is known to affect leaf morphology. Several studies have demonstrated that auxins affect leaf morphology and development (Barkoulas et al., 2008; Koenig et al., 2009). Therefore, auxins may also be related to heterophylly as a downstream target of some upstream regulators including other phytohormones. Although there is substantial evidence on the importance of auxins in leaf development, the relationship between auxin and heterophylly remains unclear. Recently some studies have demonstrated that a basic helix-loop-helix transcription factor, *PHYTOCHROME-INTERACTING FACTOR 4* (*PIF4*), regulates levels of auxin and expression of genes involved in auxin biosynthesis in response to change in ambient temperature (Franklin et al., 2011). Changes in ambient temperature are known to affect leaf morphology in *R. aquatica* (Nakayama et al., 2014). A blue light receptor protein, Cryptochrome 1 (CRY1), is reported to interact with the transcription factor PIF4 to regulate hypocotyl elongation in response to blue light (Ma et al., 2016). Blue light is one of the key environmental cues for plants under submerged conditions and is known to induce the development of submerged leaves on plants grown under the submergence (Kao and Lin, 2010). These results suggest that auxin may be one regulator of heterophylly via the transcription factors PIF4 and receptor protein CRY1, in addition to its role as a candidate for downstream target gene expression regulation.

## What is the Scope for Future Studies On Heterophylly?

Research in the past few decades has elucidated the transport processes and receptor mechanisms of various phytohormones as well as their role in various developmental processes using model plant species. These studies have demonstrated that phytohormones mutually regulate signaling and metabolic networks (Verma et al., 2016). Recent studies have identified new hormones related to the regulation of plant architecture and/or morphology (Waters et al., 2017). An interesting observation in a recent study emphasized the role of defensin-like secretory epidermal patterning factor (EPF) peptide hormones in regulating stomatal development in plants (Hara et al., 2009; Sugano et al., 2010). In many heterophyllous plants, it is known that stomatal density is altered in response to changes in the surrounding environment. Therefore, it is likely that EPF peptide hormones are also involved in the regulation of stomatal density in heterophyllous plants. These facts indicate that there is scope to study the relationships between heterophylly and the new phytohormones and their interactions with classic phytohormones for a better understanding of the phenomenon of heterophylly. Such studies will elucidate the mechanisms of acquired phenotypic plasticity, including heterophylly, through the modification of existing networks. Sequencing methods that reveal transcriptomic and epigenetic changes in response to surrounding environments have been developed during the past decade (Buenrostro et al., 2013). Additionally, a high-throughput system to measure endogenous concentration of multiple hormones, including various derivative species, has also been developed (Kojima et al., 2009). Modeling methods to integrate different levels of large-scale data and explore cause–effect relationships from the integrated data have been developed (Granier and Vile, 2014). Combination of these techniques can help to explore and understand the intricate interaction of hormones and their interactions with mechanisms of heterophylly.

Heterophylly has evolved multiple times independently during plant evolution. As expected, the mechanism of heterophylly in each plant seems to be different. For instance, heterophylly in *R. aquatica* is expressed via the KNOX-GA gene module, which regulates organogenetic activity, whereas the ethylene related pathway, which regulates cell division and elongation pattern, is reported to regulate heterophylly in *L. arcuata* (Kuwabara et al., 2003; Nakayama et al., 2014). In *P. nodosus*, the ABA-related pathway is thought to induce heterophylly (Anderson, 1978). Hence, heterophylly is an interesting model to study the convergent evolution of plant species. Although debatable, the major biological question provoked by previous studies is the implication that phenotypic plasticity can promote divergence among populations and occasionally lead to speciation (Pfennig et al., 2010). These facts signify the urgent need to study heterophylly to explore the evolution of plants as well as to understand the underlying ecological and physiological interactions.

Thus, the answer to the question “What is the scope for future studies on heterophylly?” would be to understand the relationship between heterophylly and the new hormones and the relationship between heterophylly and speciation. These studies will yield novel insights into not only the molecular mechanisms of phenotypic plasticity in plants but the evolution of plants.

## Author Contributions

All authors listed have made a substantial, direct and intellectual contribution to the work, and approved it for publication.

## Conflict of Interest Statement

The authors declare that the research was conducted in the absence of any commercial or financial relationships that could be construed as a potential conflict of interest.
